# An integrated microbiological and electrochemical approach to determine distributions of Fe metabolism in acid mine drainage-induced “iron mound” sediments

**DOI:** 10.1371/journal.pone.0213807

**Published:** 2019-03-26

**Authors:** Isabel M. Leitholf, Chrystal E. Fretz, Raymond Mahanke, Zachary Santangelo, John M. Senko

**Affiliations:** 1 Department of Geosciences, The University of Akron, Akron, OH, United States of America; 2 Department of Biology, The University of Akron, Akron, OH, United States of America; 3 Integrated Bioscience Program, The University of Akron, Akron, OH, United States of America; University of Florida, UNITED STATES

## Abstract

Fe(III)-rich deposits referred to as “iron mounds” develop when Fe(II)-rich acid mine drainage (AMD) emerges at the terrestrial surface, and aeration of the fluids induces oxidation of Fe(II), with subsequent precipitation of Fe(III) phases. As Fe(III) phases accumulate in these systems, O_2_ gradients may develop in the sediments and influence the distributions and extents of aerobic and anaerobic microbiological Fe metabolism, and in turn the solubility of Fe. To determine how intrusion of O_2_ into iron mound sediments influences microbial community composition and Fe metabolism, we incubated samples of these sediments in a column format. O_2_ was only supplied through the top of the columns, and microbiological, geochemical, and electrochemical changes at discrete depths were determined with time. Despite the development of dramatic gradients in dissolved Fe(II) concentrations, indicating Fe(II) oxidation in shallower portions and Fe(III) reduction in the deeper portions, microbial communities varied little with depth, suggesting the metabolic versatility of organisms in the sediments with respect to Fe metabolism. Additionally, the availability of O_2_ in shallow portions of the sediments influenced Fe metabolism in deeper, O_2_-free sediments. Total potential (*E*_*H*_ + self-potential) measurements at discrete depths in the columns indicated that Fe transformations and electron transfer processes were occurring through the sediments and could explain the impact of O_2_ on Fe metabolism past where it penetrates into the sediments. This work shows that O_2_ availability (or lack of it) minimally influences microbial communities, but influences microbial activities beyond its penetration depth in AMD-derived Fe(III) rich sediments. Our results indicate that O_2_ can modulate Fe redox state and solubility in larger volumes of iron mound sediments than only those directly exposed to O_2_.

## Introduction

Centuries of coal extraction in the Appalachian region of the United States has left a legacy of acid mine drainage (AMD), which remains the region’s greatest threat to surface water quality [1]. The major environmental damage caused by AMD occurs when the anoxic, acidic, and Fe(II)-rich fluid enters oxic, circumneutral streams, whereupon the higher pH enhances the oxidation of Fe(II) and precipitation of Fe(III) phases, which smother stream substrates and limit the development of robust stream ecosystems (e.g. algae, macroinvertebrates, fish; [2–4]). As such, removal of dissolved Fe(II) is the most pressing objective in AMD treatment and stream restoration activities [5]. In some cases, AMD flows as a 0.5–1 cm sheet over the terrestrial surface, resulting in aeration of the fluid and enhanced activities of Fe(II) oxidizing bacteria (FeOB; [6–11]). Continuous flow of AMD and sustained Fe(II) oxidation gives rise to massive Fe(III) (hydr)oxide deposits that are referred to as “iron mounds” or “iron terraces,” and can grow to thicknesses of meters [6, 7, 12]. While these iron mounds damage the soil and surficial systems that they cover, they may also be exploited for the treatment of AMD [6–11]. Under this scenario, the iron mounds represent iron removal systems, whereby the activities of FeOB induce oxidative removal of Fe from the AMD before the water enters nearby streams [6–11]. Notably, the iron mounds that we have encountered arise with little or no human intervention, suggesting that constructed iron mounds could serve as inexpensive and sustainable approaches to AMD treatment.

As these iron mounds grow upward, FeOB are buried in the Fe(III) (hydr)oxide phases that they produce. The dynamics of iron mound development lead to the continuous upward movement of the air-water interface, and the potential development of anoxic portions of the iron mound [12, 13]. We have noted unusual dynamics of Fe(II) oxidation and Fe(III) reduction in iron mounds, where Fe(III) reduction appears to occur in the presence of abundant O_2_, while Fe(II) oxidation might occur under conditions of severe O_2_ depletion [12, 14]. This observation may be at least partially attributable to the metabolic versatility of the acidophilic Fe-metabolizing microorganisms that inhabit the iron mounds, which are capable of Fe(II) oxidation and Fe(III) reduction (e.g. [15–18]). Indeed, the microbial communities associated with the iron mounds are remarkably uniform with depth [12]. Anaerobic activities in these iron mounds are important, because they represent a mechanism for remobilization of Fe that had been previously oxidatively precipitated—an undesirable process in the context of AMD treatment [13]. However, the distributions of anaerobic activities have proven to be difficult to predict, given their (at least partial) independence from O_2_ availability.

To assess relationships between O_2_ availability and Fe(III) reduction and microbial community dynamics associated with aerobic and anaerobic processes in an iron mound setting, we incubated initially homogenized sediments from an iron mound in North Lima, OH (referred to as the Mushroom Farm) in a column format. During these incubations we assessed Fe(II) oxidation and Fe(III) reduction, as well as the associated electrochemical signatures at discrete depths over the course of incubation in the columns. At the conclusion of the incubations, the extents of O_2_ penetration into the columns was assessed, and the microbial community composition at various depths within the columns was determined.

## Materials and methods

### Sediment collection and processing

Sediments were collected from an iron mound that developed in the sheet flow area at the Mushroom Farm in North Lima, OH with permission from Northeast Oklahoma A&M College [12, 19]. Samples were collected from the top 10 cm of the iron mound sediments using alcohol-sterilized shovels and transferred to glass jars, which were sealed for transport to The University of Akron. Sediments not immediately used were refrigerated in the dark until use. All sediments were prepared for incubations by first washing them with filter-sterilized synthetic acid mine drainage (SAMD) that contains 5 mM FeSO_4_, 5 mM CaSO_4_, 1 mM Na_2_SO_4_, 0.5 mM Al_2_(SO_4_)_3_, 0.4 mM MnSO_4_, and 0.1 mM (NH_4_)_2_Fe(SO_4_)_2_ [6]. To deactivate microbiological activity for control sediment incubations, iron mound sediments were suspended in SAMD with 3% formaldehyde [6] and incubated for 12 hours. These sediments were then washed three times with sterile SAMD to remove excess formaldehyde. The sterility of the sediments was determined by spreading the sediment-SAMD suspension on solid WAYE medium [20]. Plates were incubated in the dark at room temperature and routinely examined for growth over a period of three weeks. No growth was observed, indicating that the formaldehyde treatment effectively deactivated the sediment-associated microorganisms. After initial preparation, sediments were packed into columns as described below.

### Column construction and incubation setup

Columns were constructed using clear polycarbonate tubing (internal diameter = 5.1 cm), with ports drilled down the side of the tubing. The ports were sealed using a 1/8” strip of rubber packing on the interior of the column tube and 100% silicone sealant on the exterior. Each column was sealed at the bottom using a #11 black rubber stopper and wrapped with polyethylene tape. Prior to packing, columns were sterilized by autoclaving. Columns were packed with non-sterile or biologically deactivated sediment to a height of 108–115 mm (approximately 330 g wet sediment), and covered with 10 mm of SAMD, to mimic the water overlying the iron mound. No exogenous organic carbon was added to the sediments. Columns were conducted in triplicate and covered loosely with foil during incubation. When necessary, overlying SAMD was replenished with sterile deionized water to account for water lost to evaporation. Columns for anoxic incubations were carried out in duplicate sealed at the top with a rubber stopper, and the headspace was flushed with N_2_.

### Sampling protocol and analytical techniques

Total potential (TP) measurements were collected by inserting 13 mm x 27G Pt-Ir sub-dermal needle electrodes (Technomed-Europe; Maastricht-Airport, Netherlands) into the sampling ports arrayed along the side of the column. These electrodes were connected to a Keithley model 2000 digital multi-meter (internal resistance > 10 GΩ) with 10-channel expansion card and an Ag/AgCl reference electrode, which was placed in the SAMD at the top of the column. For anoxic incubations, the rubber stopper at the top of the columns was removed, and the headspace was continuously flushed with N_2_ while the reference electrode was inserted in the overlying SAMD. We address use of the term “total” with respect to these potential measurements in the Discussion section. Porewater samples were collected from side sampling ports using needles and syringes, and solids were removed from the liquid by centrifugation. Samples were then preserved in 0.5 M HCl before measurement of dissolved Fe(II) by ferrozine assay [21]. Theoretical *E*_*H*_ were calculated using WEB-PHREEQ [22] with the PHREEQC database [23] using the measured Fe(II) concentrations and SAMD chemistry. Since goethite was the predominant solid phase in sediments (S1 Fig), a dissolved Fe^3+^ concentration of 2.28 × 10^−3^ mM (based on the solubility of goethite at pH 1.5 in non-sterile incubations) and 4.04 × 10^−7^ (based on the solubility of goethite at pH 2.75 in biologically deactivated incubations) was assumed.

At the conclusion of the incubations, dissolved oxygen and pH measurements at 1 mm depth increments were collected using a Unisense Microsensor Multimeter Microprofiling system (version 2.01; Unisense A/S, Aarhus, Denmark); OX-N, PH-N) with Sensor Trace PRO 3.1.1 microprofiling software and pH-N (pH) and OX-N (DO) electrodes. Columns were then disassembled by cutting the polycarbonate tubing laterally and opening the half-columns. Sediments were then removed from the columns at 1 cm depth increments, and processed for further analyses. Sediment samples that were collected at this time and were intended for nucleic acid-based microbial community analysis were placed in sterile tubes and stored at -80° before DNA extraction (described below). Water content of the sediments was determined gravimetrically, and sediment-associated sulfate was determined by extracting sediment-associated and porewater sulfate with nanopure water, followed by centrifugation and quantification of sulfate in the supernatant by ion chromatography using a Dionex (Thermo Fisher Scientific Inc. Sunnyvale, CA) Basic Integrated IC System with an IonPac AS22 column and conductivity detector. Total sediment-associated carbon was quantified using a PerkinElmer 2400 Series II CHNS/O Analyzer (PerkinElmer, Inc. Waltham, MA). For x-ray powder diffraction (XRD), sediments were dried in an anaerobic chamber and analyzed using a Rigaku Ultima IV x-ray diffractometer with CuKα radiation, scanning at 2Θ of 2–70°, and accelerating voltage of 40 kV at 35 mA.

### Nucleic acid-based microbial community characterization

Before DNA extraction, Fe(III) was removed from sediments by washing with 0.3 M ammonium oxalate, with the pH adjusted to 3 with oxalic acid [24]. DNA was extracted from the Fe(III)-free material using MoBio PowerBiofilm DNA isolation kits (MoBio Laboratories, Inc. Carlesbad, CA). Partial 16S rRNA gene sequences were determined at Molecular Research LP (Shallowater, TX) by Illumina MiSeq sequencing, where 515F and 806R primers were used to amplify DNA through a 28 cycle PCR with HotStarTaq Plus Master Mix Kit (Qiagen USA, Valencia, CA) with melting at 94° for 3 min, then 28 cycles of 94° for 30 sec, 53° for 40 sec, and 72° for 1 min, followed by a 5 min elongation step at 72°. Samples were pooled and the Illumina DNA library was prepared from calibrated Ampure XP bead-purified samples. DNA was sequenced by Illumina MiSeq (San Diego, CA) following the manufacturer’s instructions. Sequences were joined and barcodes were depleted, short (<150 bp) sequences or those with unreliable base calls were removed, and the sequences were then denoised and chimeras were removed.

Sequence libraries were then further processed and analyzed in the MacQIIME environment (http://www.wernerlab.org/software/macqiime) using QIIME scripts [25]. Operational taxonomic units (OTUs) were picked de novo based on 97% sequence similarity, and assigned to taxonomic groups using the RDP classifier 2.2 with the SILVA database [26–29]. OTUs were aligned to the SILVA database using the PyNAST algorithm [30], and a phylogenetic tree was constructed. Distance matrices were constructed using the weighted UniFrac metric [31, 32], with iterative rarefaction to 68,665 sequences (75% of the number of sequences in the smallest library) with jack-knife sampling of the OTU table performed before UniFrac analysis. Unweighted pair group method with arithmetic mean (UPGMA) trees based on the produced distance matrices were then constructed using UniFrac [32]. Sequence libraries from this work have been deposited in the Sequence Read Archive under project number PRJNA490562.

## Results

### Column chemistry

**Fig 1:**
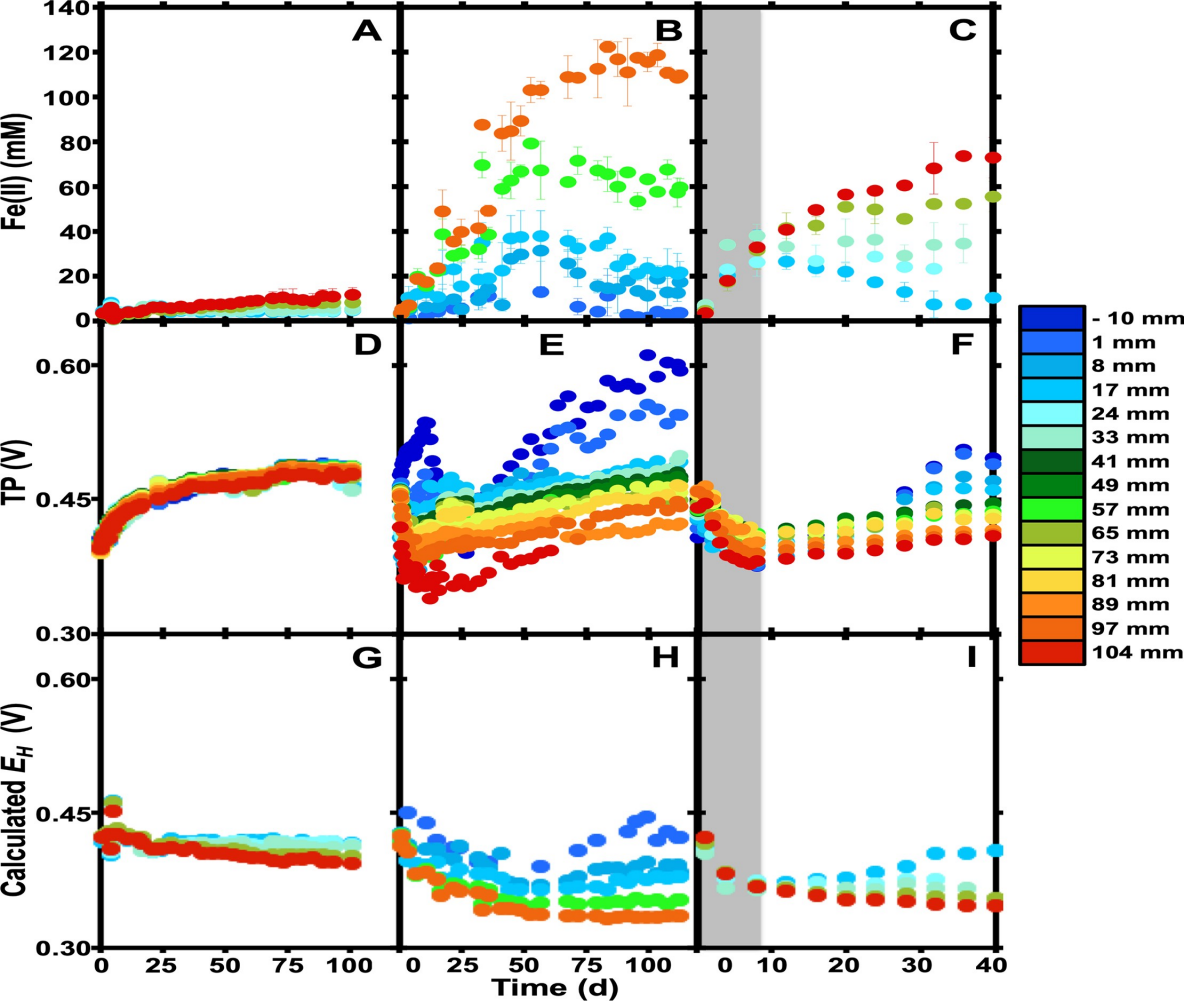
**Fe(II) concentrations (A-B), measured total potentials (TP; D-F), and theoretical *E***_***H***_ **(G-I) at various depths in column incubations containing formaldehyde-deactivated (panels A, D, and G), non-sterile continuously oxic (panels B, E, and H), and non-sterile, initially anoxic (panels C, F, and I) iron mound sediments.** Depths represent the midpoint of a depth interval of 5 mm, with the sediment-water interface at 0 mm. Gray shading in panels C, F, and I represents the time period during which the headspace of column incubations was N_2_ (with air subsequently allowed into the incubations). As a guide, cooler colors represent measurements in shallower portions of the columns, while warmer colors represent deeper portions. Error bars represent one standard deviation.

**Fig 2:**
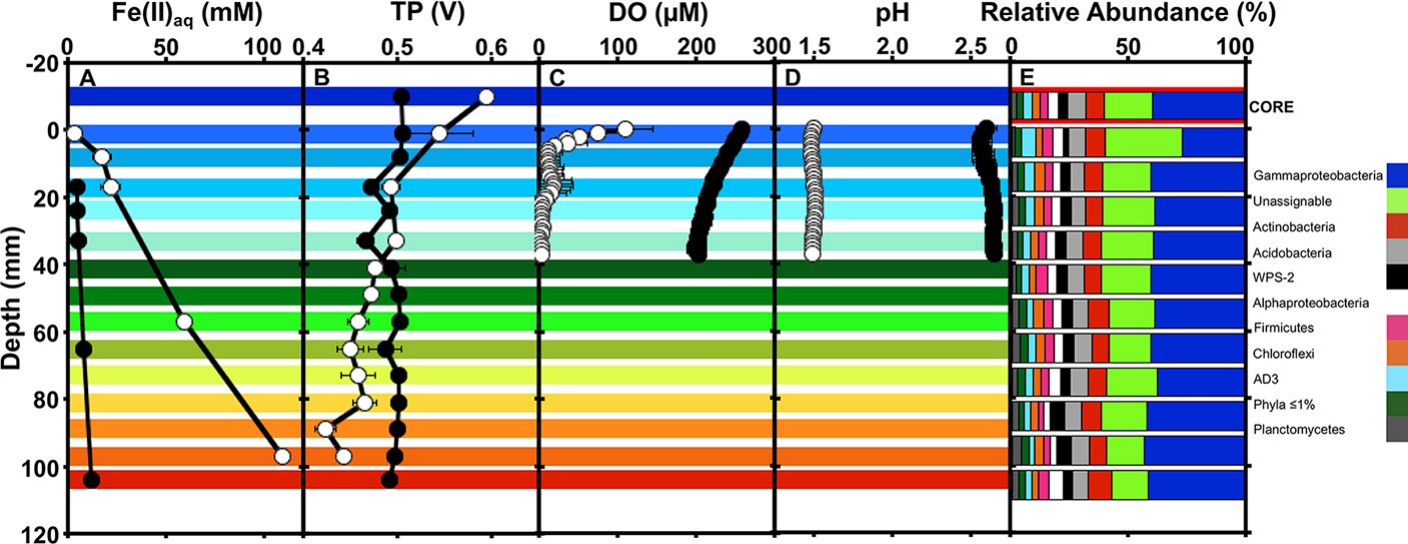
**Depth profiles of dissolved Fe(II) (Fe(II)**_**aq**_**; A), total potential (TP; B), dissolved oxygen concentrations (DO; C), pH (D), and phylum-level microbial community composition (E) at the conclusion of non-sterile (○) and formaldehyde-deactivated (●) column incubations that were incubated under continuously oxic conditions.** The colored shading across panels A-D indicates depth intervals corresponding to data points shown in Fig 1, and the color key for panel E is shown on the left side of the figure. The bar outlined in red in panel E represents the composition of the core microbiome of the entire column. Error bars represent one standard deviation.

To evaluate the dynamics of biogeochemical gradient development in iron mound sediments, columns were assembled with homogenized iron mound sediments, so that the biology and chemistry of the columns would be initially uniform throughout, and we could then visualize gradient development. In biologically deactivated sediment-containing columns, minimal Fe(II) accumulation was observed at any depth interval within the columns (Fig 1A). The observable Fe(II) accumulation may have been attributable to desorption of Fe(II) from the sediments as the sediments equilibrated with SAMD. Total potentials (TP) in inactive sediment incubations were initially lower than those of the non-sterile incubations, but approached those of the non-sterile incubations and slightly exceeded those of the theoretical *E*_*H*_ (based on the Fe^2+^/Fe^3+^ couple) after approximately 50 d (Fig 1D, 1E, and 1G). TP also remained the same with depth throughout the incubations, indicating that no electrochemical gradients developed over the course of the deactivated incubations (Figs 1D and 2B). *E*_*H*_ predicted by Fe(II) concentration were generally unchanged throughout the incubations and exhibited minor variation with depth (Fig 1G).

In non-sterile column incubations, an Fe(II) gradient developed in the first 50 d of incubation, with maximal dissolved Fe(II) concentrations of approximately 120 mM in the deepest portion of the columns due to Fe(III) reduction (Fig 1B). Early in the incubations, we observed an initial separation in TP values, with an increase in TP in the shallower sediments, and decrease in the TP of deeper sediments (Fig 1E). This split occurred concurrently with the accumulation of Fe(II) in the deeper portions of the sediments (Fig 1B and 1E). Following the initial peak in TP of shallow sediments at approximately 10 d, the TP of the shallower sediments decreased, which appears to have been due to upward diffusion of Fe(II) or reduction of easily reducible Fe(III) phases in the shallower sediments, as Fe(II) concentrations increased during this period of TP decrease (Fig 1B and 1E). TP patterns can be partially attributed to variation in Fe(II) concentration, because theoretical *E*_*H*_ patterns of shallower sediments exhibited qualitatively similar patterns to the observed TP (Fig 1E and 1H). TP of sediments below 30 mm in depth did not exhibit the increase, followed by decrease and subsequent increase in TP as was observed in the shallower sediment (i.e. ≥33 mm). Rather, the TP of these sediments decreased proportionally to their depth in the first 25 d of incubation and then gradually increased (Fig 1E). Taken together, these observations indicate that the TP is partially explained by the influence of the Fe^2+^/Fe^3+^ couple on the redox potential column porewater, but the TP is incompletely explained by *E*_*H*_.

At the conclusion of these incubations, we measured DO and pH of the upper 40 mm of the incubations (a limitation of the electrode length). In sterile incubations, the pH of the upper portion of the incubations was uniformly approximately 2.5 (Fig 2D). The initial pH of the SAMD was 3.1, and the observed decrease was likely attributable to minor amounts of abiotic Fe(II) oxidation, and hydrolysis of Fe^3+^. Any abiotic Fe(II) oxidation could be supported by O_2_ that diffused throughout the upper 40 mm of the sediments, and likely through the entire column (Fig 2A). TP and dissolved Fe(II) concentration were uniform throughout the biologically-inactive sediment columns at the conclusion of the incubations (Fig 2C). In non-sterile incubations, DO decreased in the upper 10 mm of the sediment column, and was completely depleted (detection limit 0.3 μM) below 20 mm (Fig 2C). In this portion of the column, TP decreased, and then remained nearly constant throughout the remaining depth, where the most robust Fe(III) reduction occurred (Fig 2A and 2B). This constant TP was observed despite substantial Fe(II) accumulation in the deeper, anoxic portions of the columns, which would induce lower TP (dependent on the Fe^2+^/Fe^3+^ couple; *E*_*H*_) than we observed (Fig 1E and 1H). Indeed, a linear Fe(II) gradient was observed with depth, that did not correspond to the measured TP (Fig 2A and 2B). Based on TP measurements, it appears that the upper 20 mm of the columns were where the most dramatic aerobic Fe(II) oxidation was occurring, and that activity was supported by the relatively low concentration of O_2_ that was available.

Sediment sulfate concentration (including dissolved and solid-associated) was 0.25 mmol/g (dry) and was consistent through the depth of sediments. Further, sediments were uniformly dominated by goethite throughout the columns at the conclusion of the incubations (S1 Fig), and no blackening of the sediments was observed. While we cannot rule out the possibility of cryptic sulfur cycling that supported Fe(III) reduction (whereby inorganic S species act as electron shuttles to Fe(III) [33, 34]), sulfate was not an important electron sink in comparison to Fe(III). Organic carbon (OC) concentration of the sediments was 6.5 mg/g (dry) sediment, which is consistent with OC contents of the Mushroom Farm sediments reported by Brantner et al. [12]. Organic carbon in the Mushroom Farm sediments is mostly derived from phototrophic microeukaryotic biomass and likely supported Fe(III) reduction in the sediment incubations [12]. As with sulfate concentration, OC was uniform throughout the depth of the columns at their conclusion.

To further evaluate the relationship between Fe(II) and TP, we incubated columns under anoxic conditions. During the period of anoxic incubation, Fe(II) accumulated to similar levels, regardless of depth, and TP diminished consistently with theoretical *E*_*H*_ during this period (Fig 1C, 1F and 1I). Upon introduction of atmospheric O_2_ to the headspace of the columns, Fe(III) reduction was arrested in the shallower portions of the column, but it continued in deeper portions of the column (Fig 1C). During this period of Fe(II) oxidation, the TP of shallower portions of the columns increased, and so did that of the deeper portions of the columns, despite continued Fe(III) reduction (Fig 1F). As was the case in the first set of column incubations, this increase in TP was not predicted in *E*_*H*_ based on Fe(II) concentrations (Fig 1I). Indeed, after reintroduction of atmospheric oxygen, the TP of the sediments developed similar patterns to those observed in the sediments incubated under oxic conditions throughout the incubation period (Fig 1E and 1F).

### Column sediment microbial communities

At the conclusion of the 120 d incubations, we conducted 16S rRNA gene-based surveys of microbial communities at discrete depths in the columns. Average read length of partial 16S rRNA gene sequences was 299 bp and the number of sequences recovered from each depth ranged from 91,553 to 157,653. Despite differences in activities at different depths in the column incubations, we observed little variability among the microbial communities with depth. Shannon indices of all of the depth intervals were nearly identical (5.9–6.1) (S2 Fig). Given the chemical gradients that developed over the course of the incubations, we would expect development of unique microbial communities in terms of relative abundances. This was the case to some extent, as the community in the top 10 mm could be distinguished from those of the rest of the column using the weighted UniFrac metric, but the communities from the remainder of the depths could not be distinguished from each other (S2 Fig).

When viewed compositionally at the phylum level, microbial communities were composed predominantly of Gammaproteobacteria and unassignable phylotypes as determined using the Ribosomal Database Project’s taxonomic assignment algorithm (Fig 2E).

Aside from a higher relative abundance of unassignable OTUs in the top 10 mm of the column, microbial communities varied little with depth (Fig 2E). Computation of the core microbiome (OTUs that are represented in all samples, and excluding any OTUs that are not included in all samples) in QIIME yielded a phylum-level taxonomic distribution pattern that was nearly identical to those observed in depth intervals between 10 mm and 110 mm (Fig 2E). Examination of the sequence libraries at greater taxonomic resolution revealed that 24 OTUs comprised approximately 75% of the total communities of the sediments at the conclusion of the incubations (Table 1). Even though visualizing microbial communities at the phylum level could mask variation in community composition, this was not the case in our incubations. The predominant OTUs in each phylum were consistently predominant throughout the columns. For example, the OTU attributable to Xanthomonadaceae comprised approximately 50% of the Gammaproteobacterial OTU at all depths of the column (Table 1). Nearly all of these abundant OTUs had the greatest similarity with sequences in the NCBI database that were recovered from AMD-impacted systems (Table 1), including the unassignable sequences, two of which were most similar to sequences detected in the Rio Tinto AMD system [35]. While the unassignable sequences were not similar to any cultured organisms, all but one of the OTUs with greater than 90% sequence similarity to cultivated organisms were attributable to acidophilic organisms from AMD-impacted systems, and all but three of these metabolize Fe (Table 1). Several of these Fe-metabolizing microorganisms are capable of both Fe(II) oxidation and Fe(III) reduction (Table 1). The most abundant phylotype detected in the columns was a Xanthomonadaceae, related to *Metallibacterium scheffleri*, which is an acidophilic organotrophic FeRB [36]. While *M*. *scheffleri* is not known to oxidize Fe(II), several other closely related Xanthomonadaceae-affiliated acidophiles have been shown to exploit aerobic Fe(II) oxidation for growth [37]. Our analysis of the microbial communities associated with the columns revealed relatively uniform composition of communities in sediments below 10 mm, and a narrow group of organisms (the unassigned OTUs) that were represented in greater proportions in the top 10 mm of the sediment incubations.

**Table 1:** Comparison (using BLASTn; [65]) of sequences recovered from iron mound sediment incubations to sequences contained in the GenBank database. Most closely-related 16S rRNA gene sequences from culture-independent surveys and microorganisms in culture are shown. OTUs comprising >5% of a given phylum were selected for analysis and included in the table.

Pyulum	Highest RDP-assigned taxonomy	Percentage of phylum^1^	Environment^2^	%ID^3^	Reference (acc. number)	Organism	Metabolism^4^	%ID^3^	Reference (acc. number)
Gammaproteobacteria	Xanthomonadaceae	51±3	AMD	99	[66] (JX297618)	*Metallibacterium scheffleri*	Acid/Aer/Fe(III)	98	[36] (FR874227)
	Sinobacteraceae	21±4	AMD	99	[66] (JX297610)	Bacterium A4F5	Acid/Aer/Fe(II)	100	[37] (JX869414)
	Gammaproteobacteria	10±3	AMD	99	[35] (FN862147)	*Thiohalophilus thiocyanatoxydans*	Neut/Aer	90	[77] (NR_043875
Unassigned	Unassigned	40±11	AMD	94	[35] (FN865900)	Peptostreptococcaceae AS15	Neut/Anaer	77	[78] (KX123378)
	Unassigned	20±7	AMD	96	[35] (FN866450)	*Paenibacillus tianmuensis*	Neut/Aer	78	[79] (NR_104532)
	Unassigned	18±5	geothermal	98	[67] (HF677557)	*Thermogemmatispora carboxidivorans*	Neut/Aer	86	[80] (NR_133881)
Actinobacteria	Acidimicrobiaceae	21±3	AMD	99	[68] (JQ217565)	*Acidithrix* sp. C25	Acid/Aer/Fe(II)	99	[81] (LN866582)
	Acidimicrobiales	20±3	AMD	99	[69] (FN870199)	*Aciditerrimonas ferrireducens*	Acid/Aer/Fe(III)	94	[82] (JX869415)
	Acidimicrobiales	15±1	AMD	99	[40] (KF424863)	Bacterium A4F6	Acid/Aer/Fe(II)/Fe(III)	94	[37] (JX869441)
	Acidimicrobiales	14±3	AMD	99	[70] (KC619560)	Bacterium B10H12	Acid/Aer/Fe(II)	99	[37] (NR_112972)
	Acidimicrobiales	5±1	AMD	99	[35] (FN861923)	Actinobacterium BGR 86	Acid/Aer/Fe(II)	98	[83] (GU168002)
Acidobacteria	Acidobacteriaceae	47±8	AMD	99	[71] (HG003405)	Acidobacteriaceae bacterium CH1	Acid/Aer	97	[84] (DQ355184)
	Acidobacteriaceae	38±9	AMD	99	[35] (FN866269)	*Acidipila rosea*	Acid/Aer	96	[85] (NR_113179)
WPS-2	WPS-2	46±6	AMD	99	[72] (JF737898)	*Thermosinus carboxydivorans*	Neut/Fe(III)	84	[86] (NR_117167)
	WPS-2	46±6	AMD	99	[73] (HE604029)	*Halopeptonella vilamensis*	Neut/Aer	85	[87] (NR_146012)
Alphaproteobacteria	Acetobacteraceae	55±6	AMD	99	[71] (HG003383)	Bacterium C4H7	Acid/Aer/Fe(II)	99	[37] (JX869450)
	Acetobacteraceae	16±11	AMD	99	[72] (JF737912)	*Acidisphaera* sp. PS110	Acid/Aer	97	[88] (KC954531)
Firmicutes	Alicyclobacillaceae	15±6	AMD	99	[35] (FN861437)	Alicyclobacillaceae bacterium Feo-D4-16-CH	Acid/Aer/Fe(II)/Fe(III)	93	[40] (FN870323)
	Clostridium	7±4	wastewater	96	[74] (KP717470)	*Clostridium hydrogeniformans*	Neut/Anaer	99	[89] (NR_115712)
	Alicyclobacillaceae	7±2	AMD	99	[35] (FN867136)	Alicyclobacillaceae bacterium iFeo-D4-31-CH	Acid/Aer/Fe(II)/Fe(III)	95	[40] (FN870336)
	Sulfobacillaceae	6±1	AMD	99	[75] (FN867136)	*Thermovenabulum ferriorganovorum*	Neut/Fe(III)	89	[90] (NR_042719)
Chloroflexi	Thermogemmatisporaceae	81±4	AMD	97	[76] (KP689063)	Bacterium SOSP1-79	Neut/Aer	88	[91] (AM180160)
AD3	JG37-AG-4	89±1	AMD	99	[76] (KP688954)	Bacterium B4H3	Acid/Aer/Fe(II)	99	[37] (JX869432)
Planctomycetes	Phycisphaerae	73±2	AMD	99	[35] (FN866617)	*Arenimonas maotaiensis*	Neut/Aer	81	[92] (NR_133967)

^1^Mean OTU percentage of OTU in phylum in sequence libraries from each depth interval with standard deviation of percentages from eleven depth intervals

^2^Types of environments from which sequences were recovered

^3^Percent identity based on BLASTn results

^4^Acid = acidophilic, Neut = neutrophilic, Aer = aerobic, Anaer = anaerobic, Fe(II) = Fe(II) oxidizer, Fe(III) = Fe(III) reducer

## Discussion

O_2_ availability, controlled by depth in the columns, minimally influenced the composition of microbial communities in iron mound sediments, but profoundly influenced their activities. The only phylotypes that exhibited a substantial change with depth at the conclusion of the column incubations were unassigned sequences that were similar to planktonic phylotypes observed in acidic (pH approximately 2) and high redox potential (approximately 470 mV) Rio Tinto, indicating that these organisms metabolize optimally under mostly oxic conditions [35, 38]. Otherwise, microbial communities throughout the remainder of the sediments were nearly identical (Fig 2E and S2 Fig). 16S rRNA gene-based surveys can still detect inactive organisms, which could explain the compositional similarities we have observed. In previous experiments at the Mushroom Farm, we have observed discernable shifts in microbial communities over shorter incubation times [9]. Additionally, a similar pattern of microbial community composition was observed in intact iron mound microbial communities [12]. In that case, relative abundances of phylotypes attributable to photosynthetic microeukaryotes and obligately aerobic, Fe(II) oxidizing *Gallionella* sp. diminished in deeper portions of the sediments, but other components of the microbial communities retained similar relative abundances [12]. The most notable constants in situ were Gammaproteobacteria assignable to Fe-metabolizing Xanthomonadaceae [12], which also remained abundant at the conclusion of our column incubations (Fig 2E). These observations illustrate the metabolic versatility of microorganisms with respect to Fe metabolism in AMD and AMD-impacted systems. They are often capable of Fe(II) oxidation and Fe(III) reduction, depending to some extent (but not completely) on the availability of O_2_ [14, 37, 39–41]. In the current work, we started with a homogenized microbial community from the upper 6 cm of an iron mound and challenged that community to adjust to limitations on O_2_ delivery. The communities did not vary dramatically from a compositional perspective, but exhibited dramatic differences in their activities.

Despite the consistency in community composition, the microbial activities over the course of the incubations were dramatically different at different depths, with extensive Fe(III) reduction in the deeper portions of the columns (Figs 1 and 2). O_2_ was completely depleted from the sediments at depth where Fe(III) reduction did not occur to its maximal extent (Fig 2A and 2C). In other words, the extent of Fe(II) accumulation (indicative of Fe(III) reduction) followed a gradient that was not dependent on O_2_ availability: less Fe(III) reduction was apparent at 57 mm than at 97 mm, despite complete depletion of O_2_ at 33 mm (Figs 1B and 2A and 2C). Similarly, addition of O_2_ to the initially anoxic incubations arrested Fe(III) reduction in a depth-dependent manner, and not exclusively in the shallower sediments (Fig 1C). It is not clear if Fe(II) oxidation was occurring in the anoxic sediments or if extremely low O_2_ concentrations (i.e. below the detection limit of 0.3 μM) are supporting extensive Fe(II) oxidation [42, 43]. It appears unlikely Fe(III) reduction was simply partially inhibited in the shallower sediments, because addition of air to the initially anoxic incubations led to Fe(II) oxidation in deeper sediments (Fig 1C).

Some insight into the conditions that could allow O_2_ to influence Fe(III) reduction or Fe(II) oxidation despite separation of these two species can be gained from examination of our electrochemical measurements. Electrochemical or geophysical approaches are increasingly deployed to interrogate (bio)geochemical processes in field settings and evaluate spatial distributions of microbiologically-induced redox processes [44–47]. The redox potential (*E*_*H*_) of a given solution is the potential between a non-polarizable reference electrode and a polarizable electrode in close proximity to each other and is indicative of the capacity for a solution to accept or donate electrons relative to the standard hydrogen electrode (SHE [48]). The self-potential (SP), which is widely used in geophysical surveys, represents the potential difference between two spatially-separated non-polarizable electrodes (one stationary, and one movable) and is indicative of electric current between relatively reducing and oxidizing regions or an electrochemical gradient [44, 48–52]. If a stationary non-polarizable electrode is deployed with a movable polarizable electrode, the resulting potential is referred to as total potential (TP), and represents the sum of the *E*_*H*_ and SP between the two electrodes [48]. By deploying PtIr electrodes along the column coupled with a Ag/AgCl reference electrode in the overlying SAMD, our measurements constitute the TP at various depths within the columns.

Values shown in Fig 1G–1I represent the theoretical *E*_*H*_ for the sediments based on the Fe^2+^/Fe^3+^ couple using Fe(II) concentrations from the respective experiments. It is likely that O_2_ also contributes to the *E*_*H*_ component of the TP. For instance, O_2_ was relatively high throughout the deactivated column sediments (Fig 2C), so the H_2_O/O_2_ redox couple could have influenced TP in addition to the Fe^2+^/Fe^3+^ couple. Indeed, TP were higher than predicted based on the Fe^2+^/Fe^3+^ redox couple (Fig 1D and 1G). However, at the DO in the deactivated columns at the conclusion of the incubations, the theoretical *E*_*H*_ was 0.96 V. Therefore, while O_2_ clearly contributed to TP in regions where it was present, it appears that the Fe^2+^/Fe^3+^ redox couple exerted the most control on the *E*_*H*_ component of TP throughout the columns. Additionally, since we could not detect evidence of sulfate reduction in these incubations, the Fe^2+^/Fe^3+^ redox couple would predominantly drive *E*_*H*_ in the sediments. The measured TP do not necessarily constitute *E*_*H*_, since the non-polarizable and polarizable electrodes are spatially separated from each other, but the calculated *E*_*H*_ (based on Fe(II) concentration) and measured TP of the formaldehyde-deactivated incubations match reasonably well (Fig 1D and 1G), as do the *E*_*H*_ and TP during anoxic incubations (gray-shaded part of Fig 1F and 1I). Qualitatively, in both of the non-sterile incubations, shallower sediments, with lower Fe(II) concentrations and greater rates and extents of Fe(II) oxidation exhibited higher TP and *E*_*H*_ (Fig 1B, 1C, 1E, 1F, 1H and 1I and Fig 2A and 2B). The higher *E*_*H*_ is consistent with lower Fe(II) concentration, and perhaps higher dissolved Fe^3+^ concentration. For instance, the higher-than-predicted TP in overlying AMD and shallow sediments (Fig 1E and 1H) could be attributable to accumulation of Fe^3+^ exceeding its maximum solubility in the oxic portions of the columns where Fe(II) oxidation is most robust. Overall, these observations illustrate the contribution of redox potential, as controlled by the Fe^2+^/Fe^3+^ redox couple, to the TP measured in these incubations.

*E*_*H*_ calculations did not predict the continuous increase in TP at all depths in the incubations after approximately 20 d (Fig 1E and 1H). They also did not predict the increase in TP upon addition of O_2_ to initially anoxic incubations (Fig 1F and 1I). If based exclusively on Fe(II) concentration, these observed TP would predict a decrease in Fe(II) concentration, which was not the case. In fact, Fe(II) concentrations segregated further with depth as the TP increased (Fig 1B and 1E). An explanation for the observed increase in TP in anoxic incubations could be opposing gradients of Fe^2+^ and Fe^3+^, where a high rate of Fe(II) oxidation in shallow, oxic portions of the column induced the Fe(II) gradients that we observed, while downward diffusion of Fe^3+^ from the oxic zone to the oxic zone induced the increase in TP. However, the rate of Fe(III) reduction during the anoxic period of the incubations (3.7 mM/d, based on Fe(II) accumulation) exceeded the rate of Fe(II) oxidation in the shallowest sediments (0.97 mM/d, based on Fe(II) depletion) after O_2_ was allowed into the columns (Fig 1F). This pattern of Fe(II) oxidation and Fe(III) reduction rates would result in a steep Fe(II) gradient near the oxic-anoxic interface, and not the gradual Fe(II) gradient from the top to the bottom of the column that we observed here (Fig 2). While TP and predicted *E*_*H*_ closely matched during the anoxic period of the short-term incubations, upon introduction of O_2_ to the headspace, the TP increased at all depths, and Fe(II) concentrations segregated based on depth in the columns (Fig 1C, 1F and 1I). These inconsistencies between the TP and *E*_*H*_ when O_2_ is available at the top of the columns can be attributed to the SP contribution to TP [48], and suggest an electron transfer process occurring in the sediments due to the electrochemical pull of O_2_ overlying the sediments.

Both field- and laboratory-scale electrochemical/geophysical surveys of SP signals have illustrated the development of SP signals across regions that connect high and low *E*_*H*_ regions as we have observed here [45, 51, 52]. In order to facilitate the electron transfer that gives rise to SP signals in sediments, it has been suggested that a perhaps disorderly, but integrated network of microorganisms, extracellular material, and redox-active solid phases gives rise electron transfer [45, 52, 53]. Such a model could function quite well in iron mound settings, as the sediments are composed almost exclusively of Fe(III) (hydr)oxide phases [6, 7, 10, 12], and these phases could facilitate the electron transfer processes [54–64], with opposing Fe(II) and O_2_ concentration gradients providing the driving force for electron transfer.

Our results are consistent with previous field and laboratory observations of a gap between intrusion of O_2_ into the sediments and the zone of Fe(II) oxidation, where an O_2_ intrusion front and Fe(II) oxidation zone were spatially separated [12, 14]. This work has allowed us to visualize the microbially-mediated development of these gradients in the iron mound sediments and apply electrochemical approaches to assess biogeochemical processes within the sediments. Our results indicate that the chemical and microbiological influence of O_2_ in iron mound sediments exceeds its actual penetration into the sediments. Notably, Fe(II) accumulation in deeper sediments was suppressed despite no O_2_ availability. If engineered iron mounds are to be used for oxidative precipitation and removal of Fe(II) from AMD [8–10], our results indicate that the longer range influence of O_2_ into the sediments could minimize reductive re-release of Fe(II) from the sediments.

## Supporting information

S1 Fig Powder X-ray diffraction patterns of sediments collected from columns at the conclusion of non-sterile 120 d incubations. The reference diffraction pattern of goethite in the top panel is from The American Mineralogist Crustal Structure Database [Downs TR, Hall-Wallace M. Am Mineral 2003; 88:247–250.].
(DOCX)

S2 Fig PCoA of microbial communities associated with different depths of iron mound sediment incubations at the conclusion of the incubations (120 d) using the weighted and Unifrac metric [Lozupone CA,Hamady M, Kelly ST, Knight R. Appl Environ Microbiol 2007; 73: 1576–1585.**].** Values in parentheses in depth legend indicate Shanon Indices of microbial communities at each of those depths. Values in parentheses of axis labels indicate the percentage of variation explained by a PCo.
(DOCX)
