# Supplementary material for: An integrated microbiological and electrochemical approach to determine distributions of Fe metabolism in acid mine drainage-induced “iron mound” sediments
Source: PLoS One. 2019 Mar 26;14(3):e0213807. doi: 10.1371/journal.pone.0213807 (PMC6435174; doi:10.1371/journal.pone.0213807)
Supplement: S1 Fig — The reference diffraction pattern of goethite in the top panel is from The American Mineralogist Crustal Structure Database [Downs TR, Hall-Wallace M. Am Mineral 2003; 88:247–250.]. (DOCX) [file pone.0213807.s001.docx]

**
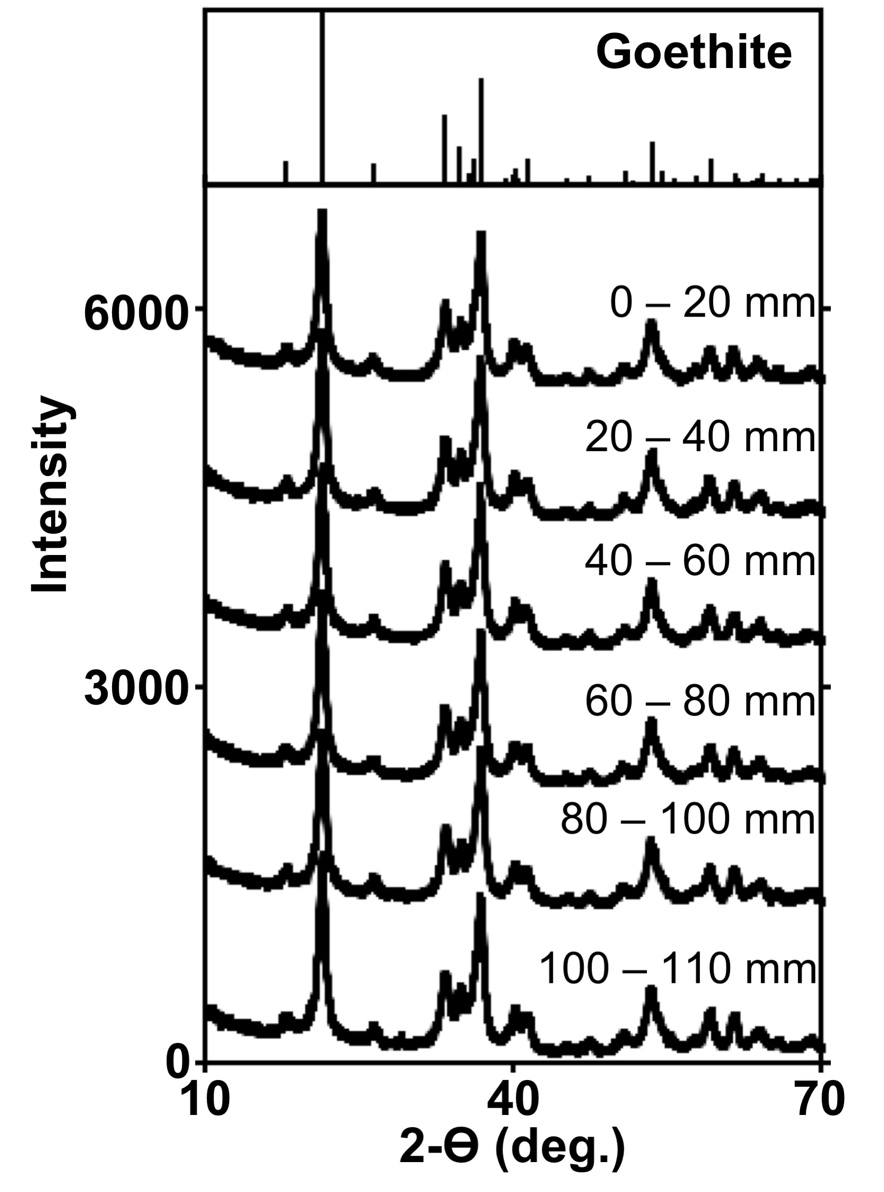
**

**S1 Fig.** Powder X-ray diffraction patterns of sediments collected from columns at the conclusion of non-sterile 120 d incubations. The reference diffraction pattern of goethite in the top panel is from The American Mineralogist Crustal Structure Database (91).
