# Supplementary material for: An integrated microbiological and electrochemical approach to determine distributions of Fe metabolism in acid mine drainage-induced “iron mound” sediments
Source: PLoS One. 2019 Mar 26;14(3):e0213807. doi: 10.1371/journal.pone.0213807 (PMC6435174; doi:10.1371/journal.pone.0213807)
Supplement: S2 Fig — Appl Environ Microbiol 2007; 73: 1576–1585.]. Values in parentheses in depth legend indicate Shanon Indices of microbial communities at each of those depths. Values in parentheses of axis labels indicate the percentage of variation explained by a PCo. (DOCX) [file pone.0213807.s002.docx]

**
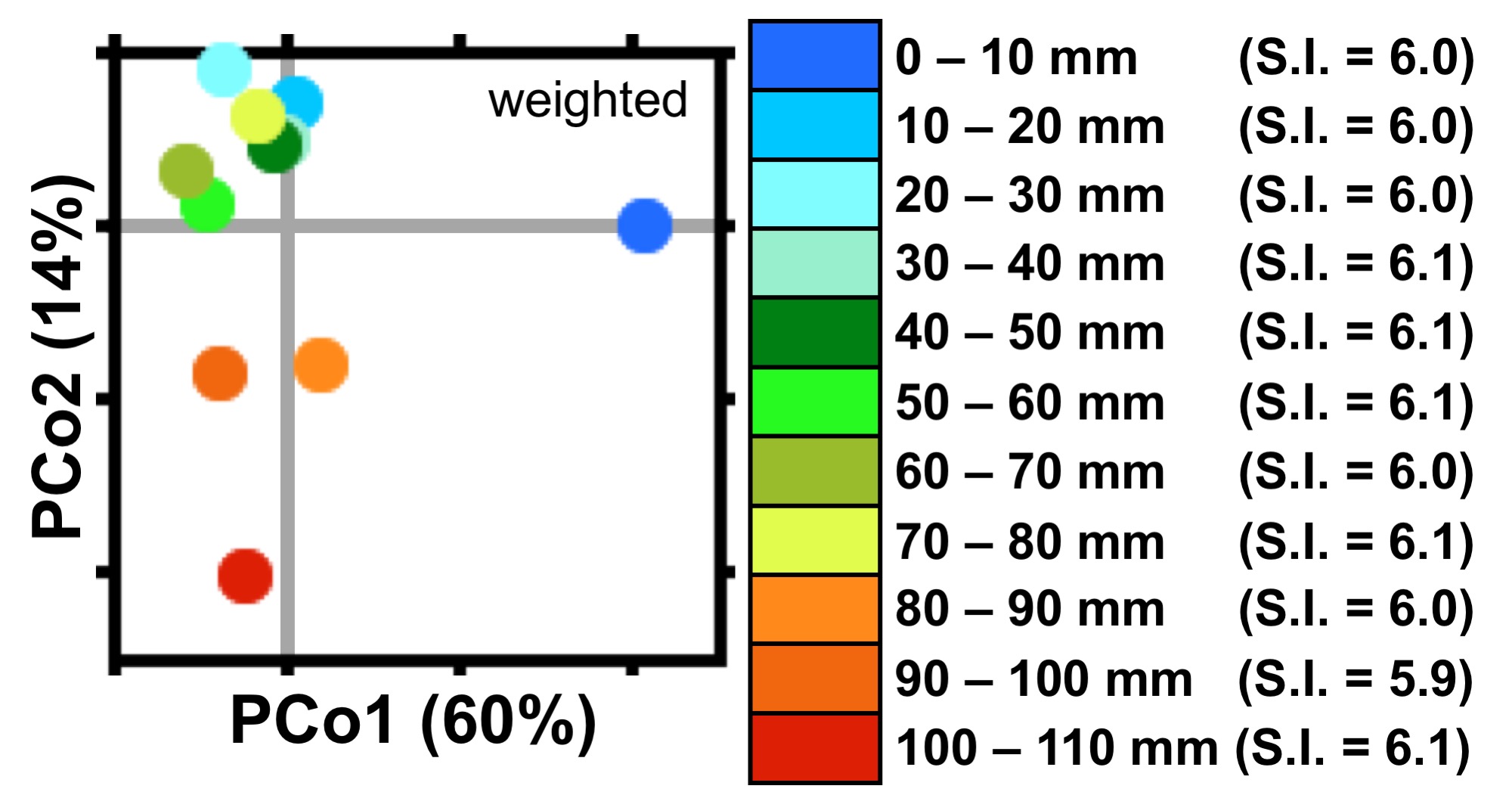
**

**S2 Fig.** PCoA of microbial communities associated with different depths of iron mound sediment incubations at the conclusion of the incubations (120 d) using the weighted and Unifrac metric (30). Values in parentheses in depth legend indicate Shanon Indices of microbial communities at each of those depths. Values in parentheses of axis labels indicate the percentage of variation explained by a PCo.
